# Effectiveness of Different Web-Based Interventions to Prepare Co-Smokers of Cigarettes and Cannabis for Double Cessation: A Three-Arm Randomized Controlled Trial

**DOI:** 10.2196/jmir.3246

**Published:** 2014-12-05

**Authors:** Julia Becker, Severin Haug, Robin Sullivan, Michael Patrick Schaub

**Affiliations:** ^1^Swiss Research Institute for Public Health and Addiction ISGFUniversity of ZurichZurichSwitzerland

**Keywords:** tobacco, cannabis, co-smoking, simultaneous cessation, motivational enhancement, personalized feedback, web-based intervention, motivational interviewing

## Abstract

**Background:**

The relationship between tobacco and cannabis use is strong. When co-smokers try to quit only one substance, this relationship often leads to a substitution effect, that is, the increased use of the remaining substance. Stopping the use of both substances simultaneously is therefore a reasonable strategy, but co-smokers rarely report feeling ready for simultaneous cessation. Thus, the question of how co-smokers can be motivated to attempt a simultaneous cessation has arisen. To reach as many co-smokers as possible, we developed brief Web-based interventions aimed at enhancing the readiness to simultaneously quit tobacco and cannabis use.

**Objective:**

Our aim was to analyze the efficacy of three different Web-based interventions designed to enhance co-smokers’ readiness to stop tobacco and cannabis use simultaneously.

**Methods:**

Within a randomized trial, three brief Web-based and fully automated interventions were compared. The first intervention combined the assessment of cigarette dependence and problematic cannabis use with personalized, normative feedback. The second intervention was based on principles of motivational interviewing. As an active psychoeducational control group, the third intervention merely provided information on tobacco, cannabis, and the co-use of the two substances. The readiness to quit tobacco and cannabis simultaneously was measured before and after the intervention (both online) and 8 weeks later (online or over the phone). Secondary outcomes included the frequency of cigarette and cannabis use, as measured at baseline and after 8 weeks.

**Results:**

A total of 2467 website users were assessed for eligibility based on their self-reported tobacco and cannabis co-use, and 325 participants were ultimately randomized and analyzed. For the post-intervention assessment, generalized estimating equations revealed a significant increase in the readiness to quit tobacco and cannabis in the total sample (B=.33, 95% CI 0.10-0.56, *P*=.006). However, this effect was not significant for the comparison between baseline and the 8-week follow-up assessment (*P*=.69). Furthermore, no differential effects between the interventions were found, nor were any significant intervention or time effects found on the frequency of tobacco or cannabis use.

**Conclusions:**

In the new field of dual interventions for co-smokers of tobacco and cannabis, Web-based interventions can increase the short-term readiness to quit tobacco and cannabis simultaneously. The studied personalized techniques were no more effective than was psychoeducation. The analyzed brief interventions did not change the secondary outcomes, that is the frequency of tobacco and cannabis use.

**Trial Registration:**

International Standard Randomized Controlled Trial Number (ISRCTN): 56326375; http://www.isrctn.com/ISRCTN56326375 (Archived by WebCite at http://www.webcitation.org/6UUWBh8u0).

## Introduction

### The Relationship Between Tobacco and Cannabis Use

Although smoking tobacco is the leading global cause of preventable death [1], cannabis is the most widely used illicit drug [2] and is associated with a range of physical and mental health problems [3,4]. Both substances are often used together; the majority of cannabis users also smoke cigarettes. In a study in the United States, 74% of marijuana users smoked cigarettes, compared to 29% of nonusers [5]. Furthermore, cannabis use is reportedly more common among cigarette smokers than it is among nonsmokers. In the National Survey on Drug Use and Health in the United States, the 30-day prevalence of cannabis use was 38% among tobacco smokers and only 11% among nonsmokers [6]. In a similar survey in Switzerland, cannabis use in the previous 12 months was reported by 28% of adolescents who smoked tobacco daily compared to 9% and 2% of the adolescents who were ex-smokers and never-smokers, respectively [7].

The mechanisms that link the use of both substances are assumed to go beyond the mechanisms that explain the co-use of drugs in general [8]. For instance, both substances are usually smoked (have a shared route of administration) and are often used simultaneously (co-administration), that is, tobacco is added to cannabis joints (“mulling”) or is smoked directly after cannabis (“chasing”) [8,9]. In Switzerland, 97% of cannabis users smoke cannabis joints mixed with tobacco [10].

In the context of cessation, the relationship between the substances is often problematic. For instance, tobacco smokers who also consume cannabis seem to make fewer efforts to quit tobacco [11] and tend to be less successful in quitting tobacco than tobacco-only smokers [12]. Furthermore, the cessation of one substance is frequently accompanied by an increased use of the other [13-15], and cessation programs that exclusively address tobacco appear to be less effective for co-smokers of cannabis [16,17].

### Interventions for Tobacco and Cannabis Use

Despite these findings, interventions have typically targeted tobacco or cannabis use alone and have rarely addressed both substances simultaneously. One explanation for the separate treatments may be that in many industrialized countries, the treatment of cannabis dependence is provided by psychiatrists, whereas interventions for tobacco smokers are part of a more general public health system [18,19]. However, the body of literature on the relationship between tobacco and cannabis use is growing, and authors of recent reviews perceive a demand for double interventions that target tobacco and cannabis simultaneously [8,9,20]. In line with this notion, a preliminary study of the development of such a program has indeed revealed this demand [21]. The experts and the co-smokers who participated in the preliminary study considered a dual cessation intervention to be feasible.

However, the participants also expected only modest readiness to simultaneously quit tobacco and cannabis use; half of the surveyed co-smokers were unaware of the association between tobacco and cannabis use [21]. Due to this finding, the authors developed three brief online interventions to enhance co-smokers’ awareness of the relationship between the substances and their readiness to simultaneously quit each substance. The purpose of the current study was to evaluate these online interventions and examine how co-smokers’ readiness to simultaneously quit tobacco and cannabis can be augmented. Motivational constructs such as the stages of change and the readiness to quit have been shown to predict the subsequent engagement in interventions [22,23] and abstinence [24].

Because of its easy access and ubiquitous presence, the Internet arose as a potentially effective medium to reach a large number of co-smokers who might be unaware of the relationship between their tobacco use and their cannabis use. Personalized, normative feedback is one motivational technique that can be applied to Web-based interventions for substance use. Based on the social norms approach [25], such interventions typically include self-assessment sections and feedback sections in which the participants’ behavior is compared to a reference sample. The overestimation of substance use by others is common and is positively associated with one’s own use [26]. Web-based social norm interventions use this association and aim to correct the participants’ erroneous perceptions. Primarily recruiting college students and targeting alcohol use, Web-based norms approaches for interventions have yielded promising results [27].

Another established technique for building motivation is motivational interviewing (MI), which uses a client-centered, directive counseling style to explore and reduce ambivalence and increase the intrinsic motivation for change [28]. Brief face-to-face interventions based on MI have been found to be effective in reducing cannabis use [29] and may assist in smoking cessation [30]. MI in Web-based interventions is usually applied as a chat-intervention but is not fully automated. However, the first promising results of fully automated MI have recently been revealed by a computer-based intervention that targets perinatal drug use [31].

For this study, we developed three Web-based interventions that apply the above-mentioned techniques, that is, normative feedback and MI. For an active control group, we used Web-based psychoeducation. In addition, to maintain the low threshold for Internet access and keep the study dropout rate as low as possible, the interventions were designed as brief single-session interventions.

### Aims of the Study and Hypotheses

The main aim of this study was to evaluate the effectiveness of three Web-based interventions to enhance co-smokers’ readiness to quit both tobacco and cannabis simultaneously. Our first hypothesis (H1) was that the tested interventions would be effective in enhancing the readiness to simultaneously quit tobacco and cannabis use. Thus, we assumed a significant within-subjects effect for assessment time. Because particular interactive interventions that were tailored to individuals have shown promising effects in aiding smoking cessation [32], our second hypothesis (H2) was that interactive and tailored interventions, that is, an intervention based on MI and an intervention that provides normative feedback, would more effectively enhance co-smokers’ readiness to quit tobacco and cannabis use simultaneously than would mere psychoeducation. Because MI has shown promising effects as a motivational enhancement strategy for cannabis users [33], we additionally hypothesized that this intervention would outperform the effectiveness of the normative feedback intervention (H3).

Furthermore, this study aimed to evaluate the three interventions as they pertained to secondary outcome variables, that is, the frequencies of tobacco and cannabis use. We had the same hypotheses for these outcomes as those explained above.

## Methods

### Study Design and Setting

To test our hypotheses, we conducted a three-armed randomized trial with pre-, post- and 8-week follow-up assessments. (ISRCTN56326375; see Multimedia Appendix 1 [34] for the CONSORT EHEALTH checklist.)

The three single-session interventions were Web-based and fully automated. The baseline assessment (t0) was conducted at the beginning of the intervention session, and the post-intervention assessment (t1) immediately followed the intervention. After 8 weeks, the subjects were re-assessed (follow-up, t2) by phone or online. However, our primary focus was set on the immediate post-intervention assessment because our primary outcome, the readiness to simultaneously quit tobacco and cannabis, would not be applicable to participants who stopped smoking tobacco or cannabis after the intervention at t2. The data were collected between January and November 2012.

The interventions were integrated within the German-language website of the program “i-cut”. In addition to the interventions, the website contained information about an integrative group cessation course for co-smokers of tobacco and cannabis. This cessation course is evaluated in a separate study (ISRCTN15248397).

**Figure 1:**
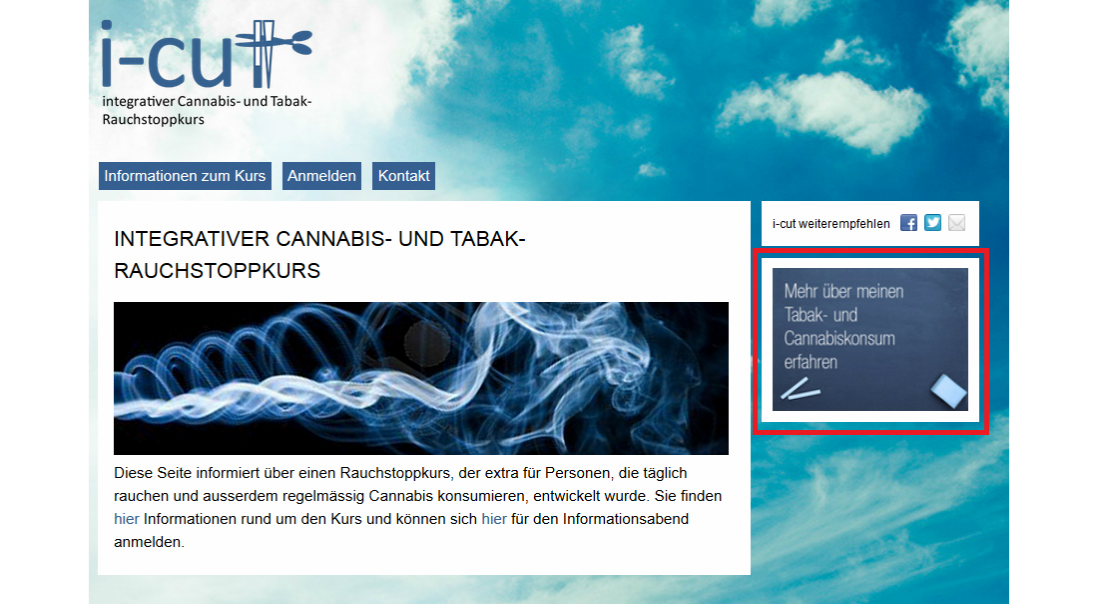
Screenshot of the teaser (red square) for the Web-based intervention as displayed on the course information pages.

**Figure 2:**
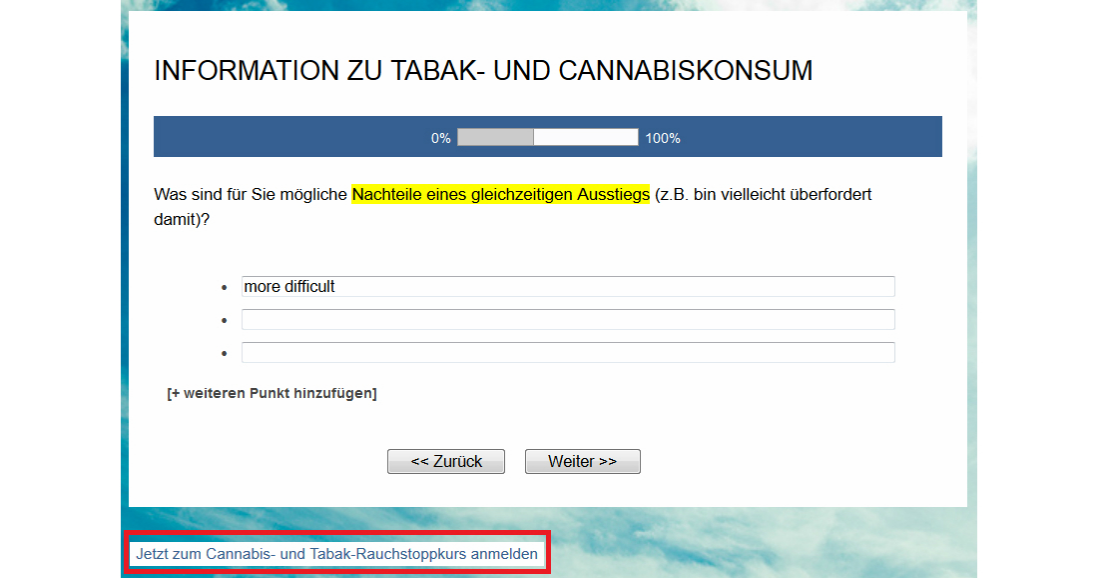
Screenshot of the intervention (intervention arm: motivational interviewing) and the hyperlink (red square) that directed participants to the Web pages with information about the smoking cessation course.

Participants could enter the current study in one of two ways. First, they could enter it directly from the start page of the website, where participants could choose between “getting more information about the course” and “learning more about my use of tobacco and cannabis”. They were then directed to the course information pages or to the intervention session, respectively. We chose the cover term “learning more about my use of tobacco and cannabis” for the intervention session to attract co-smokers who were not seeking treatment. The second way to enter the study was to switch there from the course information pages by clicking a teaser that was displayed on the right side of each information page. It was also labeled “learning more about my use of tobacco and cannabis” (Figure 1). Conversely, participants could switch from the intervention session to the course information pages by clicking a hyperlink (“register now for the tobacco and cannabis cessation course”). This hyperlink was present on every page of the intervention, and the participants who clicked on it were directed to the course information pages and dropped out of the present study. Figure 2 shows a sample page of the intervention and the hyperlink.

### Eligibility and Recruitment

The inclusion criteria for study participation included any tobacco use during the past 4 weeks and any cannabis use during the past 6 months. As implicit inclusion criteria, participants had to speak German and be computer literate. There were no age restrictions or other exclusion criteria.

Recruitment for the present study ran parallel to recruitment for a feasibility study of the above-mentioned smoking cessation course for co-smokers of tobacco and cannabis. This was conducted in Zurich and a neighboring city. The recruitment strategy has been described in the publication on the course development [21]. Briefly stated, recruitment was carried out online and offline. First, a press release about the course was issued, which resulted in several reports in local newspapers and on radio and TV stations. Furthermore, brochures and pamphlets were sent to counseling centers for addiction prevention and treatment, psychiatrists, and health (care) centers in the canton of Zurich and in the bordering cantons. Additionally, two social media platforms and a teaser in the online edition of a popular free newspaper were used for recruitment. All of these referred readers to the start page of the website for more information.

To maximize the response rates, study participants were also offered the opportunity to participate in a lottery for three vouchers valued at 300, 200, or 100 Swiss Francs after they completed the first session, including the second measurement. Additionally, a second lottery for the same values served as an incentive to participate in the follow-up measurement. The data were collected from January to December 2012.

### Procedure

**Figure 3:**
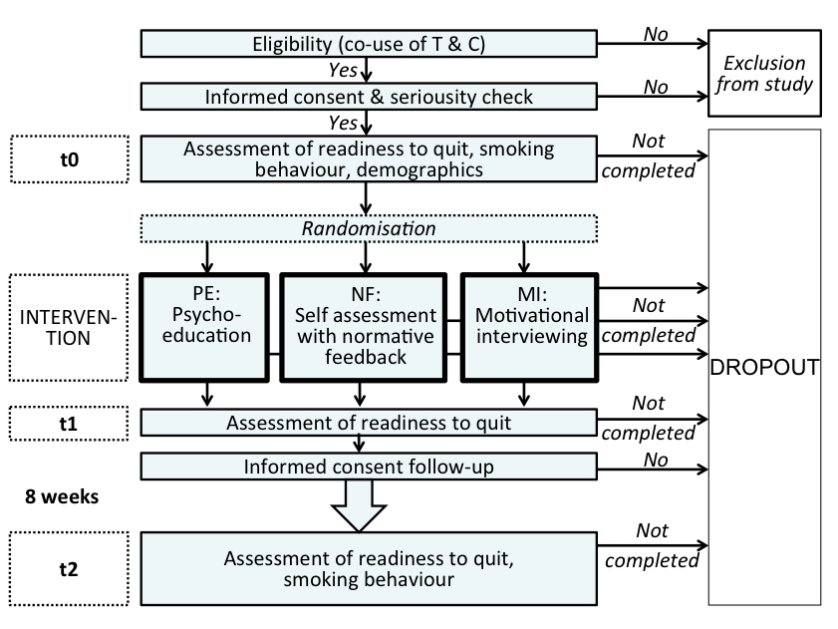
Study procedure.

Figure 3 illustrates the study procedure in detail. The initial questions presented to potential participants were used to check the inclusion criteria. If the users met these criteria, they were informed about the opportunity to participate in a study that aimed to improve the website’s information offerings. Those who did not meet the inclusion criteria or did not provide informed consent were excluded from the study and were referred to a webpage that contained information on tobacco and cannabis use, which was also provided in the psychoeducational intervention (see below). The study participants were then instructed to create an anonymous but personal identification code that combined certain letters of their parents’ names and their own date of birth. The same procedure was applied at the follow-up assessment to link the data of the different assessments.

Once the baseline measurement (t0) was completed, participants were randomly assigned to one of three possible interventions. After finishing the intervention, participants were reassessed (post-intervention, t1) and informed about the 8-week follow-up assessment (t2). To keep the threshold for entering the study as low as possible, the information about the follow-up assessment was provided only at this point. This was done because the main aim of the present study was to enhance the readiness to simultaneously quit tobacco and cannabis between t0 and t1. Participants who provided informed consent for the follow-up assessment could indicate whether they wanted to answer the follow-up questionnaire online or over the phone. At the end of the session, participants were referred to the webpage of the group cessation program if they were interested.

For the follow-up assessment, participants were contacted after 8 weeks via their chosen medium (ie, via an email that included a link to the online questionnaire or via telephone). Those who preferred to answer the questionnaire online received an email reminder after approximately 2 weeks if they had not yet completed the online questionnaire. Those who chose the telephone questionnaire were contacted up to ten times.

### Interventions

#### General Information and Technological Background

Participation in the interventions was free, and access was open for every eligible participant. The delivery of interventions was fully automated. An open source software, LimeSurvey (Version 1.91), was used to program the survey and the interventions. As described below, the interventions varied in the extent to which they were interactive with the participants. Additionally, the interventions differed in the way in which each was tailored to the responses that the participants had given during the baseline assessment and during the interventions themselves.

#### Intervention 1: Normative Feedback

The first intervention contained a combination of self-assessment and personalized normative feedback (NF). It consisted of three sections that included one each for tobacco use, cannabis use, and co-smoking. In the first and second sections, participants began by completing a questionnaire: the Fagerstrom Test of Nicotine Dependence (FTND) [35,36] and the Cannabis Use Disorder Identification Test (CUDIT) [37], respectively. Participants received feedback following each questionnaire. Feedback was individually tailored to participants using an algorithm based on the results from the FTND, the CUDIT, and the baseline data. Based on the social norms approach, each participant’s reported frequency of smoking was presented in relation to the normative data from Swiss community samples. Afterwards, participants received feedback about their questionnaire scores and were told whether their responses met the criteria for dependency (FTND) and/or problematic use (CUDIT), respectively. Explanations of “cigarette dependence” and “problematic cannabis use” were also given. Each substance-specific section concluded with brief recommendations for cessation or moderation of use. In addition, at the end of the intervention, information was provided that simultaneously accounted for the participant’s use patterns of tobacco and cannabis. Participants who regularly smoked both tobacco and cannabis were informed about the group cessation course and referred to the end of the post-intervention assessment for further information. Participants who used either just one of the substances or both less regularly received contact details for the appropriate consulting services. Table 1 presents examples of translated feedback.

**Table 1:** Examples of feedback provided during the normative feedback intervention to a participant who smoked more than five cigarettes per day and used cannabis less than once per week.

Intervention step	Example
Feedback on tobacco use frequency	You indicated smoking an average of 12 cigarettes per day. Among Swiss males, 70% do not smoke at all. Only approximately 10% smoke more than you.
Feedback on cigarette dependence	Your nicotine dependence is classified as high. Your result means that quitting may be more difficult for you compared to people with low dependence. Presumably, you will experience withdrawal symptoms. Nevertheless, these symptoms will weaken soon, and there are helpful aids against them. For instance, nicotine replacement therapy is very effective. However, quitting smoking requires more than just getting through the withdrawal symptoms. For example, you should develop individual strategies to help you cope with risk situations where the temptation of smoking a cigarette is high. Professional support (eg, a smoking cessation course) can be very helpful in developing such strategies.
Feedback on cannabis use frequency	During the past 4 weeks, you used cannabis two or three times. A survey revealed that 89% of Swiss adolescents and young adults do not use cannabis at all. Only 4% use it more often than you.
Combined feedback	Of course, it is not easy to quit both substances simultaneously for good, especially after having smoked cigarettes on a regular basis. You can ask for support at [name of a center for addiction counseling and treatment] and mention that you also smoke joints occasionally.

#### Intervention 2: Motivational Interviewing

The second intervention was based on the principles of motivational interviewing (MI). It was highly interactive and tailored to the participant, and it used a selection of MI techniques that could be adapted to a Web-based intervention, such as open-ended questions, affirmative feedback, and periodic summaries. The aim of this intervention was to promote participants’ self-reflective thinking about their own smoking behavior and intentions to change it and to enhance their self-confidence in the ability to change. This was done through different tasks, such as decisional balance tasks, in which participants wrote down personal pros and cons of stopping tobacco use, cannabis use, or both simultaneously (Figure 2). Participants were also asked to write down what advice they would give to a co-smoking friend and to indicate their confidence in successfully stopping tobacco, cannabis, or both simultaneously on a confidence ruler. Participants received feedback, including a brief summary of their indicated change in self-confidence and a brief informational text about the simultaneous cessation of tobacco and cannabis use. To further enhance their self-confidence, participants were asked to list any behavior that they had successfully changed in the past and to write down the names of persons in their network who could provide some level of social support during their attempt to quit smoking. Participants who, at baseline, had low levels of motivation to quit smoking and cannabis simultaneously received an additional task.

#### Intervention 3: Psychoeducation

**Figure 4:**
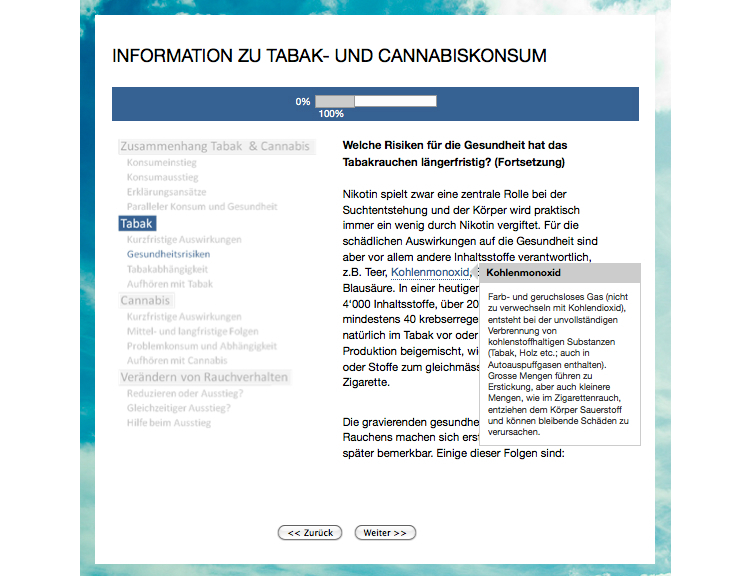
Screenshot of a section in the psychoeducational intervention.

The third intervention was the active control group. It provided psychoeducational information (PE) about tobacco and cannabis use. The information was thematically subdivided into smaller subsections. Participants had to read the sections in sequential order. Several terms and concepts that some readers may not know (eg, “carbon monoxide”) were explained in a small text box that appeared when mousing over the word of interest (Figure 4). The PE intervention started with an explanation of the association between the two substances with regard to the initiation and cessation of their use, their linking mechanisms, and the potential health consequences of their co-use. The next chapter contained information about the short-term and long-term consequences of tobacco use, tobacco dependence, and the cessation of tobacco use and was followed by an analogous chapter on cannabis. The final chapter provided information about changing smoking behavior and addressed smoking reduction versus abstinence, the simultaneous cessation of tobacco and cannabis use, and support during the cessation process. At this point, the group cessation program was mentioned, and participants were referred to the end of the post-intervention assessment to receive further information.

### Outcome Measures

The primary outcome measure was participants’ readiness to quit the use of tobacco and cannabis simultaneously. Readiness was measured at all three time points by the question, “To what extent are you ready to quit tobacco and cannabis simultaneously?” Participants indicated their readiness on a ruler ranging from 1 (“not at all”) to 10 (“very much”). The item was designed based on the contemplation ladder [22], which is especially suited to measure the early stages of readiness. In addition, a comparison of the readiness ruler to other measures of motivation to change revealed its good concurrent and predictive validity and its superior clinical utility when its brevity and ease of administration are considered [38].

Secondary outcomes included the self-reported frequency of tobacco and cannabis use at baseline (t0) and at the 8-week follow-up (t2). The frequency of tobacco use was defined as the daily amount of cigarettes smoked during a typical smoking day, corrected for the number of smoking days during the past month. The frequency of cannabis use in the prior week was assessed using a 7-day timeline follow-back question [39].

### Baseline Measures

At baseline, we assessed key demographic variables: age, sex, highest educational attainment, and nation of residence. Furthermore, we measured participants’ smoking history: age of onset of tobacco and cannabis use and the number of prior attempts to quit tobacco use, cannabis use, or both simultaneously.

### Statistical Analysis

According to the intention-to-treat principle, all participants who provided informed consent and communicated their intention to provide serious answers to the questionnaire were included in the analyses. Using the Amelia II multiple imputation package of the R software environment for statistical computing, Version 2.15.3 [40], we imputed 20 datasets. In a simulation study using data from an online self-help program for problem drinkers, Amelia II outperformed other methods of multiple imputation [41]. Hypotheses tests were performed using each dataset separately and were pooled afterwards (intention-to-treat analysis). To determine the robustness of our results to the analytic strategy, we also performed complete case analyses considering only participants who provided data at all three assessments.

The trial arm differences in the baseline measurements were tested using analysis of variance (ANOVA) and the Kruskal-Wallis test for continuous variables and chi-square tests for categorical variables, depending on each variable’s parametric properties. We conducted dropout analyses for the post-intervention and the follow-up assessment using logistic regression analyses.

To analyze the primary and secondary outcome variables, we used generalized estimating equations (GEE) that considered the correlated nature of repeated measures. In the GEE models used to test H1, the only predictor was measurement time. The GEE models used to test differential effects of the interventions (H2 und H3) considered five variables: measurement time, intervention type, interaction of time and intervention type, gender, and baseline readiness to quit cannabis. Measurement time was entered as a dummy-coded categorical variable with the baseline measurement as the reference category. In the GEEs that modeled the secondary outcomes, time was binary because the frequency of use was measured only at t0 and t1. Interaction effects examined whether the intervention type had a differential effect on the changes in the outcome variable over time in the two compared groups, thus answering H2 and H3. Gender and baseline readiness to quit cannabis use were included as control variables because they differed between the two groups at baseline.

To directly test the postulated differential effects by intervention group, the interventions were grouped by hypothesis and analyzed in two separate models. One model contrasted the PE intervention as a reference category with the combination of MI and NF (H2), whereas the second model contrasted the two interactive, personalized intervention types, NF and MI, with each other (H3).

All GEE models were based on an unstructured working correlation matrix. For the models of readiness to quit, a normal model with an identity link function was chosen. In the models of frequency of tobacco and cannabis use, we used a negative binomial model with a log link function. In case of statistically significant results, Cohen’s *d* was calculated. The alpha level was set at alpha=.05, and the analyses were performed using R [40], Stata 12.1 SE [42], IBM SPSS Statistics Version 20 [43], and G*Power 3.1.3 [44].

The power calculation was based on the outcome of primary interest, the readiness to simultaneously cease tobacco and cannabis use, as measured directly after the intervention (t1). The study was powered to detect a small effect because most reviews of Web-based interventions that aim at changing tobacco or cannabis use behavior report small intervention effects [45,46]. For a 3 intervention arms x 2 repeated measurements design, a total sample size of N=246 was required when assuming a small effect size of f=.10, according to Cohen [47], with a 2-sided, type I error rate alpha=.05, and a power of 80%.

### Ethical Approval

The study was designed in accordance with the Declaration of Helsinki and was approved by the ethics committee of the Canton of Zurich, Switzerland (approval number: KEK-StV-Nr. 23/11, June 27, 2011, and amendment for the Internet-based intervention, November 11, 2011).

## Results

### Baseline Characteristics, Response Analysis, and Intervention Duration

**Figure 5:**
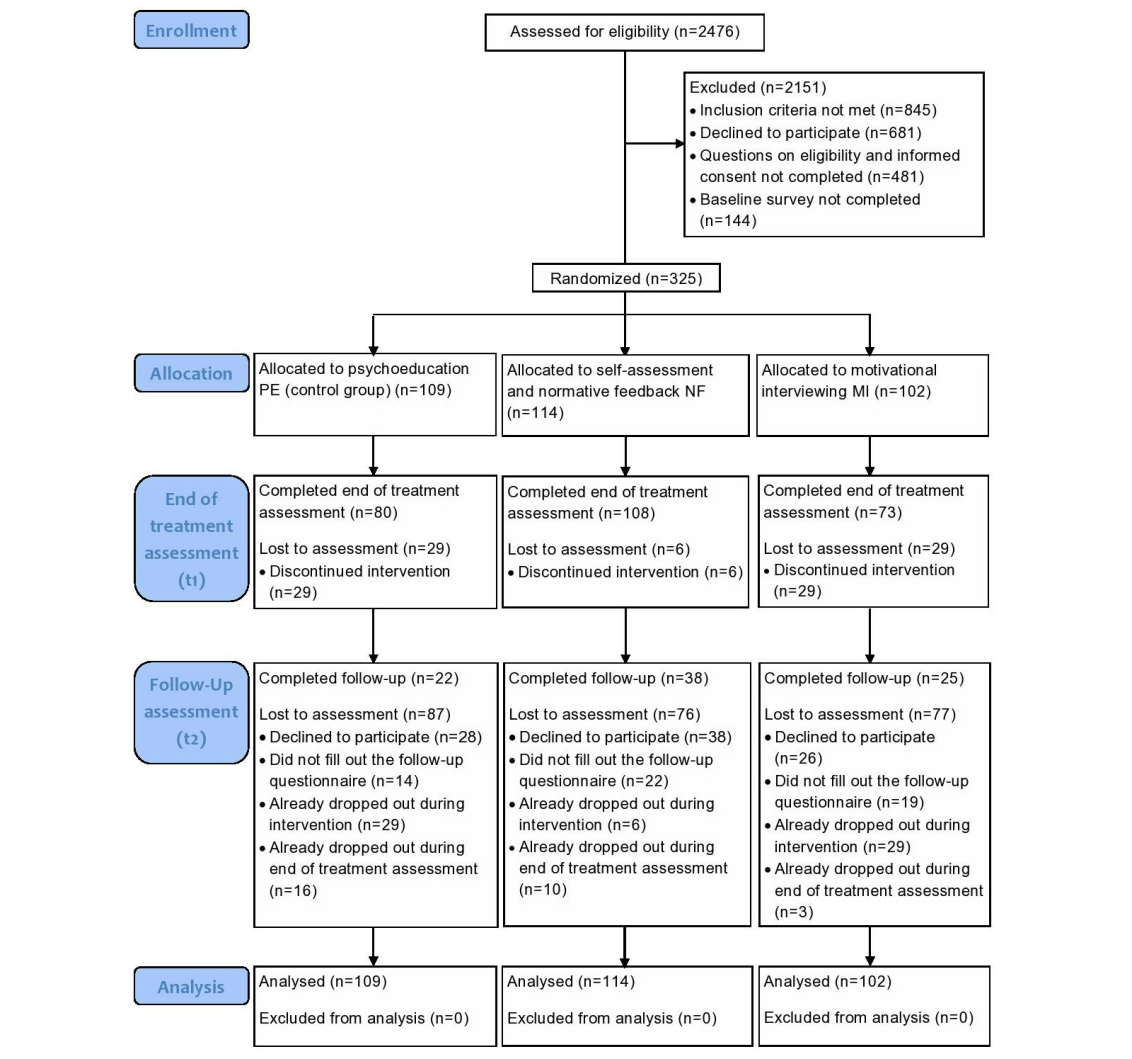
Study flow chart.

As shown in Figure 5, 1631 of the 2476 users who were assessed for eligibility met inclusion criteria. Of those, less than a quarter (325/1631, 19.93%) provided informed consent and completed the baseline assessment and could therefore be randomized into one of the intervention groups. Of them, 80.3% (261/325) of participants completed the intervention and participated in the post-intervention assessment, and 26.2% (85/325) participated in the follow-up assessment.

Table 2 compares the baseline variables across the intervention groups. Except for the relatively high percentage of women (30.3% in the PE intervention vs 14.9% in the NF intervention and 17.6% in the MI intervention) and the higher readiness to quit cannabis use in the PE intervention, no baseline variables differed significantly across the three groups.

During the intervention and the post-intervention measurement (t1), 28.6% (93/325) of participants dropped out, and 28.3% (92/325) did not provide informed consent for the follow-up assessment. Of the 85 follow-up participants, 51 (60%) responded via the online questionnaire and 34 (40%) were followed up with via phone. The dropout analysis revealed that participation in the post-intervention assessment was predicted by the intervention condition and the readiness to stop tobacco and cannabis simultaneously at baseline. A lower readiness to stop simultaneously at baseline predicted a higher probability of participating in the post-intervention assessment: OR 0.89, 95% CI 0.80-0.988, SE 0.05, *P*=.03. Furthermore, participants in the NF condition had a participation rate of 94.7% and thus were significantly more likely to answer the post-intervention questions than the PE (73.4%) and the MI conditions (71.6%) (OR 6.32, CI 2.49-16.00, SE 0.47, *P*<.001). Participation at the follow-up assessment was predicted by sex, that is, males had a lower likelihood of participating at the follow-up than did females (OR 0.49, 95% CI 0.24-0.98, SE 0.36, *P*=.04). In contrast to the post-intervention assessment, the intervention groups did not significantly differ regarding their participation at the follow-up.

The duration of the interventions differed significantly. Overall, participants remained in the intervention sessions for an average of 25.5 minutes (SD 33.0), including the baseline and post-intervention assessments. Whereas the participants in the NF condition finished the session after a mean of 17.0 minutes (SD 9.1) on average, participants in the PE (mean 28.4, SD 38.4) and the MI (mean 28.9, SD 41.6) interventions stayed significantly longer (*F*_2,322_=4.7, *P*=.01). However, there were no significant associations between the intervention duration and the outcomes, that is, the readiness to stop tobacco and cannabis simultaneously at the post-intervention assessment (*r*=.08, *P*=.22), the readiness to stop tobacco and cannabis simultaneously at the follow-up (*r*=.06, *P*=.60), tobacco use frequency at the follow-up (*r*=.15, *P*=.20), or cannabis use frequency at the follow-up (*r*=-.08, *P*=.49).

**Table 2:** Trial arm differences in baseline variables.

	PE (n=109)	NF (n=114)	MI (n=102)	Significance	
				*F*(df) / χ^2^(df)	*P*
Females, n (%)	33 (30.3)	17 (14.9)	18 (17.6)	8.91 (2)	.01
Age in years, mean (SD)	30.5 (9.5)	29.2 (9.6)	29.6 (9.5)	1.22 (2)	.54
Tobacco use frequency (cigarettes per day), mean (SD)	12.5 (7.7)	12.0 (8.2)	13.6 (8.6)	2.16 (2)	.34
Cannabis use frequency (times per day), mean (SD)	2.5 (1.9)	2.3 (2.3)	2.3 (2.2)	3.54 (2)	.17
Age of tobacco use onset in years, mean (SD)	16.0 (3.2)	15.8 (2.9)	16.0 (2.7)	0.17 (2,322)	.84
Age of cannabis use onset in years, mean (SD)	17.1 (4.4)	16.3 (3.4)	16.5 (3.1)	1.09 (2,322)	.34
Prior simultaneous cessation attempt, n (%)	32 (29.4)	38 (33.6)	32 (31.4)	0.47 (2)	.79
Readiness to quit tobacco, mean (SD)	7.2 (2.4)	7.0 (2.7)	7.5 (2.4)	1.42 (2)	.49
Readiness to quit cannabis, mean (SD)	5.8 (3.0)	4.8 (3.3)	5.1 (2.9)	6.03 (2)	.049
Readiness to quit tobacco and cannabis simultaneously, mean (SD)	5.2 (2.8)	4.7 (3.0)	5.0 (2.9)	2.40 (2)	.30

### Effects of the Intervention on Readiness to Simultaneously Quit Tobacco and Cannabis Use

**Figure 6:**
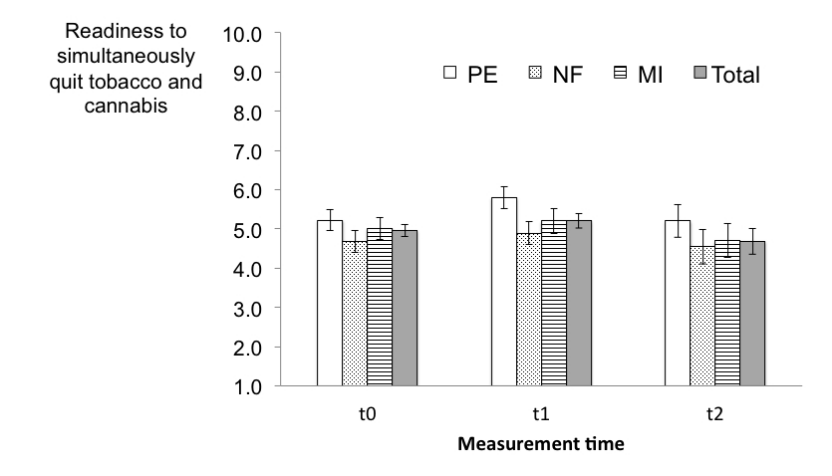
Course of readiness to simultaneously quit tobacco and cannabis use over time, pooled means of the intention-to-treat sample, including imputations; error bars represent the standard error of the mean (PE=psychoeducation, NF=normative feedback, MI=motivational interviewing).

As shown in Figure 6, the readiness to quit tobacco and cannabis use simultaneously slightly increased in all interventions between t0 and t1 and decreased thereafter. The GEE analysis used to test time effects in the total sample (H1) revealed that readiness to simultaneously quit was significantly higher at post-intervention than at baseline (B=.33, SE 0.12, 95% CI 0.10-0.56, *P*=.006). The effect size for the mean difference between these two assessments was small (*d*=0.20). At the follow-up assessment, the readiness to simultaneously quit was no longer significantly higher than at the baseline level (B=-.13, SE 0.33, 95% CI -0.81-0.54, *P*=.69).

Table 3 displays the results of the GEE models that tested the two hypotheses related to the differential change in readiness to quit tobacco and cannabis use simultaneously. Regarding H2, there was a significant main effect of time in examining the change in readiness to quit simultaneously from baseline (t0) to the post-intervention (t1) assessment. The effect size for this time effect, now based on the reference group PE, was small (*d*=0.38). This effect was not maintained at follow-up (t2). Furthermore, neither the intervention effect nor the time*intervention interaction was significant. As the analysis of H3 revealed, there were no significant time effects of readiness to quit simultaneously when only MI and NF were included in the model. In both models, the control variable baseline readiness to stop cannabis use at baseline was a significant predictor of readiness to stop both tobacco and cannabis use simultaneously.

The complete case analyses replicated these findings. The first model, which used the total sample, revealed a significant time effect at t1 (B=.31, SE 0.11, 95% CI 0.37-1.54, *P*=.001) but not at t2 (*P*=.17). Regarding H2, only the time effect observed when comparing the post-intervention with the baseline assessment was significant (B=.95, SE 0.18, 95% CI 0.37-1.54, *P*=.001). In the model used to test H3, there was no significant effect, except for the control variable baseline readiness to quit cannabis use.

**Table 3:** Results from the linear GEE models (with 20 imputed datasets) that examined readiness to quit tobacco and cannabis use simultaneously, according to H2 and H3.

Hypothesis	Parameter	B	Standard error	95% CI		*P*
				lower	upper	
H2	Intercept	1.83	0.38	1.07	2.58	<.001
	Groups NF & MI^a^	0.07	0.25	-0.43	0.56	.80
	Time t2^b^	-0.06	0.42	-0.89	0.78	.90
	Time t1^b^	0.59	0.19	0.22	0.96	.002
	Time t2^b^× Groups NF & MI^a^	-0.12	0.40	-0.90	0.66	.76
	Time t1^b^× Groups NF & MI^a^	-0.40	0.22	-0.83	0.03	.07
	Baseline readiness to stop cannabis use	0.57	0.04	0.48	0.65	<.001
	Female gender^c^	0.17	0.29	-0.40	0.74	.56
H3	Intercept	1.59	0.45	0.70	2.47	<.001
	Group MI^d^	0.20	0.29	-0.37	0.76	.50
	Time t2^b^	-0.03	0.42	-0.88	0.81	.94
	Time t1^b^	0.22	0.14	-0.06	0.50	.13
	Time t2^b^× Group MI^d^	-0.30	0.45	-1.18	0.58	.50
	Time t1^b^× Group MI^d^	-0.06	0.28	-0.60	0.48	.83
	Baseline readiness to stop cannabis use	0.59	0.05	0.49	0.69	<.001
	Female gender^c^	0.29	0.41	-0.50	1.09	.47

^a^reference: PE.

^b^reference: Time t0 (baseline).

^c^reference: male gender.

^d^reference: NF.

### Effects of the Intervention on Secondary Outcomes

Descriptive statistics for the frequency of tobacco and cannabis use showed only a weak decrease in frequency between the baseline and follow-up assessments (Table 4). The GEE model that analyzed H1 did not reveal a significant time effect for the frequency of either tobacco use (Incidence Risk Ratio [IRR] -0.04, SE 0.09, 95% CI -0.23-0.15, *P*=.70) or cannabis use (IRR -0.05, SE 0.12, 95% CI -0.29-0.20, *P*=.70).

None of the analyses of differential changes in the frequency of either tobacco or cannabis use revealed a significant time effect, intervention effect or time*intervention interaction.

The complete case analyses predominantly replicated these findings, revealing no significant time, group, or time*group interaction effects in the GEE models of tobacco or cannabis use frequency. One exception was a significant time effect for the frequency of tobacco use among the total sample (H1: IRR 0.89, SE 0.04, 95% CI 0.81-0.98, *P*=.02).

**Table 4:** Means and standard deviations of the frequency of tobacco and cannabis use at baseline (t0) and 8-week (t2) follow-up (descriptive statistics were calculated using the 20 imputed datasets).

Outcome variable	Time points	PE, mean (SD)	NF, mean (SD)	MI, mean (SD)	Total
Tobacco use frequency, cigarettes per day	t0	12.5 (2.4)	12.0 (2.5)	13.6 (2.5)	12.7 (2.5)
	t2	12.5 (2.5)	11.0 (2.5)	13.4 (2.7)	12.3 (2.6)
Cannabis use frequency, times per week	t0	2.5 (1.4)	2.3 (1.4)	2.3 (1.4)	2.3 (1.4)
	t2	2.4 (1.4)	2.2 (1.4)	2.2 (1.4)	2.2 (1.4)

## Discussion

### Principal Results

This study evaluated three brief fully automated Web-based interventions that aimed to enhance co-smokers’ readiness to simultaneously quit their tobacco and cannabis use. Regarding the readiness to simultaneously quit using tobacco and cannabis, we assumed that all participants would have an increased level of readiness after the intervention compared to the baseline assessment (H1). Furthermore, we hypothesized that more interactive and individually tailored interventions would be more effective than mere information provision (psychoeducation; H2). Additionally, we tested the hypothesis that a Web-based intervention that applies principles derived from MI would be even more effective than an intervention that provides tailored, normative feedback (H3). The hypotheses regarding tobacco and cannabis use frequency were analogous.

Regarding the readiness to simultaneously quit tobacco and cannabis use, the results supported our first hypothesis. That is, in the total sample, the readiness to simultaneously quit was significantly elevated at the post-intervention assessment relative to baseline. This effect had disappeared by the 8-week follow-up assessment. The two hypotheses that assumed differential intervention effects were also not supported. With regard to the frequency of tobacco and cannabis use, our analyses did not reveal time or intervention effects.

### Strengths and Limitations

Among the strengths of this study is that it is the first study of Web-based interventions that target co-smokers of tobacco and cannabis. Furthermore, the interventions are fully automated and therefore require no personnel beyond their initial development.

Among the limitations of this study was its high attrition rate regarding participation at the follow-up assessment. However, high attrition rates are common in eHealth studies and brief interventions [48]. We addressed this limitation by using multiple imputation methods and performing traditional complete case analyses. Furthermore, our primary focus was set on the post-intervention assessment, which had a lower attrition rate. However, the dropout analyses revealed that participants dropped out selectively before the post-intervention assessment; that is, participants of the NF-condition were more likely to complete the post-intervention assessment, indicating the attrition superiority of normative feedback to the other two conditions. More difficult to explain is the finding that participants with a lower baseline readiness to quit simultaneously were more likely to complete the post-intervention assessment. A possible explanation of this finding might be that lower baseline readiness to quit simultaneously resulted in more uncertainty when confronted with the interventions. Possibly, this uncertainty resulted in a greater interest in the interventions and made participants following the intervention for a longer time than those participants that were already more ready for simultaneous cessation. Regarding the follow-up assessment, there was only one significant predictor of participation: female sex. However, this variable was statistically controlled in the GEE analyses because the proportion of males and females also differed between the intervention conditions.

A further limitation is that we did not include an assessment-only control group and could therefore not control for baseline assessment effects. We did not include such a control group because the baseline assessment, the intervention, and the post-intervention assessment happened in the same session. Therefore, individuals in an assessment-only control group would have been reassessed after less than 30 minutes.

Finally, the NF and MI interventions differed in length. The intervention sessions for MI and PE participants both lasted nearly 30 minutes, but the duration of an NF intervention session was approximately 11 minutes shorter. This difference was also reflected in the higher participation rate among NF participants in the post-intervention session. The possibility that we would have achieved significant differences between these two interventions if they had been equally long can therefore not be excluded.

### Comparison With Prior Findings

The comparability to prior studies is limited because no Web-based interventions that target the co-use of tobacco and cannabis have been published. Additionally, Web-based MI interventions that are delivered fully automated and do not use chat-based MI-counseling are rare. However, the significant time effect in our study and the absence of differential intervention effects on readiness to quit are in line with the findings of a study that compared a single-session of MI-based chat-intervention with a chat in which participants received technical information about the baseline self-test [49]. That study included problematic alcohol and cannabis users but targeted only the particular problem behavior. The interventions were comparable to the MI and PE interventions of the current study with regard to their length but differed from the current interventions because they were not delivered in an automated fashion.

Moreover, we speculate that providing knowledge was a relatively effective measure in our study because co-smokers’ baseline knowledge about the relationship between tobacco and cannabis use seems to be generally modest [21]. In addition, the psychoeducational intervention was the only intervention that provided information on the risk of physical harm from cannabis use. In one previous study, awareness of this risk was a significant predictor of readiness to simultaneously quit [21].

There are several possible explanations for the lack of time effects on the frequency of tobacco and cannabis use. First, the interventions were conceptualized as motivational enhancement interventions and targeted co-smokers who were in earlier stages in the process of behavior change. The interventions therefore had mainly motivational contents and only very few elements that are commonly applied to support the cessation or reduction of tobacco or cannabis use, such as the development of personal strategies or skills training. It has been previously shown that the effectiveness of Internet interventions in creating behavior change is associated with the incorporation of behavior change techniques [50]. In addition, the studied interventions were very brief compared to Web-based treatment interventions, which revealed significant effects on either tobacco use [51,52] or cannabis use [53,54]. This explanation is supported by the fact that other studies that analyzed Web-based interventions of a comparable length also revealed no effect on cannabis use [49,55]. It should also be considered that the current interventions targeted two behaviors simultaneously, which may require especially intensive interventions. Finally, the high attrition rate limits the interpretability of our findings concerning behavior change. This limitation is also illustrated by the different findings from the intent-to-treat and the complete case analysis.

Moreover, the appropriateness of fully automated MI is questionable because some components of the MI approach, such as therapeutic rapport, cannot be realized in an online setting. The efficacy of fully automated MI might be particularly limited when two behaviors are targeted simultaneously because dual cessation presumably provokes ambivalence that cannot be counterbalanced by a therapist. However, in brief face-to-face interventions for universal drug prevention and early intervention, MI was also not more effective than advice [56,57]. Compared to our study, however, significant changes over time in tobacco, cannabis, and alcohol use were achieved in both intervention groups. Furthermore, a Web-based intervention to promote smoking cessation using seven 45-minute sessions with MI-based video-chat in a virtual reality world revealed both significant time and intervention effects but used an assessment-only control condition [51].

### Conclusions

The findings of this study suggest that brief fully automated Web-based interventions have a short-term but perhaps no longer-term effect on co-smokers’ readiness to simultaneously quit tobacco and cannabis use. There were no differential intervention effects, indicating that psychoeducation is not less effective compared to more individualized, interactive interventions when the co-use of tobacco and cannabis is targeted. Moreover, neither time nor intervention effects on substance use behavior were found. For dual-health behavior change, more intensive interventions regarding the length and the mode of administration (fully automated vs face-to-face, text-chat, or video-chat) may be needed. Future studies could examine more comprehensive Web-based treatment interventions for co-smokers and examine the efficacy of chat-based MI-counseling in this target group.

## Multimedia Appendix 1

CONSORT EHEALTH checklist V1.6.1 [57].

## Abbreviations

CUDIT: Cannabis Use Disorder Identification Test
FTND: Fagerstrom Test of Nicotine Dependence
GEE: generalized estimating equations
MI: motivational interviewing (intervention 2)
NF: normative feedback (intervention 1)
PE: psychoeducation (intervention 3)
SE: standard error
t0: baseline assessment
t1: post-intervention assessment
t2: 8-week follow-up assessment
