# Supplementary material for: Effectiveness of Different Web-Based Interventions to Prepare Co-Smokers of Cigarettes and Cannabis for Double Cessation: A Three-Arm Randomized Controlled Trial
Source: J Med Internet Res. 2014 Dec 5;16(12):e273. doi: 10.2196/jmir.3246 (PMC4275498; doi:10.2196/jmir.3246)
Supplement: Supplementary file 1 [file jmir_v16i12e273_app1.pdf]

CONSORT-EHEALTH Checklist V1.6.2 Report

(based on CONSORT-EHEALTH V1.6), available at [http://tinyurl.com/consort-ehealth-v1-6].

3246

Date completed

1/13/2014 11:46:32

by

Julia Becker

Effectiveness of different web-based interventions to prepare co-smokers of cigarettes and cannabis for double-cessation: A three-arm randomised controlled trial

TITLE

1a-i) Identify the mode of delivery in the title

"web-based interventions"

1a-ii) Non-web-based components or important co-interventions in title

Not applicable: All components of the interventions are web-based. Only the follow-up assessemnt was partially carried out via phone.

1a-iii) Primary condition or target group in the title

"co-smokers of cigarettes and cannabis"

ABSTRACT

1b-i) Key features/functionalities/components of the intervention and comparator in the METHODS section of the ABSTRACT

"Within a randomised trial, three brief, web-based and fully automated interventions were compared. The first intervention combined the assessment of cigarette dependence and problematic cannabis use with personalised, normative feedback. The second intervention was based on principles of motivational interviewing. As an active psycho-educational control group, the third intervention merely provided information on tobacco, cannabis, and the co-use of the two substances."

1b-ii) Level of human involvement in the METHODS section of the ABSTRACT

"three brief, web-based and fully-automated interventions"

1b-iii) Open vs. closed, web-based (self-assessment) vs. face-to-face assessments in the METHODS section of the ABSTRACT

"web-based and fully automated interventions"
"was measured before and after the intervention (online), as well as eight weeks later (online or over phone)"

1b-iv) RESULTS section in abstract must contain use data

"A total of 2467 website-users were assessed for eligibility based on their self-reported tobacco and cannabis co-use, and 325 participants were ultimately randomised and analysed. "

1b-v) CONCLUSIONS/DISCUSSION in abstract for negative trials

"The studied personalised techniques were no more effective than was psychoeducation."
Reasons for the lack of differential intervention effects are discussed in the dicussion section of the article.

INTRODUCTION

2a-i) Problem and the type of system/solution

"While smoking tobacco is the leading global cause of preventable death [1], cannabis is the most widely used illicit drug [2] and is associated with a range of physical and mental health problems [3,4]. Both substances are often used together, as the majority of cannabis users also smoke cigarettes. "
"The mechanisms that link the use of both substances are assumed to go beyond the mechanisms that explain the co-use of drugs in general [8]. "
"In the context of cessation, the relationship between both substances is often problematic.
"Despite these findings, interventions have typically targeted tobacco or cannabis use alone and have rarely addressed both substances simultaneously."
"a preliminary study of the development of such a program has indeed revealed this demand [21]. The experts and the co-smokers who participated in the preliminary study considered a dual cessation intervention as feasible.
However, the participants also expected only modest readiness to simultaneously quit tobacco and cannabis use, as half of the surveyed co-smokers were unaware of the association between tobacco and cannabis use [21]. Due to this finding, we developed three brief online interventions to enhance co-smokers' awareness of the relationship between the substances as well as their readiness to simultaneously quit each substance. "

2a-ii) Scientific background, rationale: What is known about the (type of) system

"Personalised, normative feedback is one motivational technique that can be applied to web-based interventions for substance use. Based on the social norms approach [25], such interventions typically include self-assessment sections and feedback sections in which the participants' behaviour is compared to a reference sample. The overestimation of substance use by others is common and is positively associated with one's own use [26]. Web-based social norm interventions use this association and aim to correct the participants' erroneous perceptions. Mostly studied among college students and targeting alcohol use, web-based norms approaches for interventions have yielded promising results [27]. "
"A further established technique for building motivation is motivational interviewing (MI), which uses a client-centred, directive counselling style to explore and reduce ambivalence and increase the intrinsic motivation for change [28]. Brief face-to-face interventions based on MI have been found to be effective in reducing cannabis use [29] and may assist in smoking cessation [30]. MI in web-based interventions is usually applied as a chat-intervention but is not fully automated. However, the first promising results of fully automated MI have recently been revealed by a computer-based intervention targeting perinatal drug use [31]."
"For an active control group, we used web-based psychoeducation. "

METHODS

3a) CONSORT: Description of trial design (such as parallel, factorial) including allocation ratio

"The main aim of this study was to evaluate three web-based interventions regarding their effectiveness in enhancing co-smokers' readiness to quit both tobacco and cannabis simultaneously. Our first hypothesis (H1) was that the tested interventions would be effective in enhancing readiness to simultaneously quit tobacco and cannabis use. Thus, we assumed a significant within-subjects effect for assessment time. Because particular interactive interventions that were tailored to individuals have shown promising effects in aiding smoking cessation [32], our second hypothesis (H2) was that interactive and tailored interventions, i.e. an intervention based on MI and an intervention providing normative feedback, would more effectively enhance co-smokers' readiness to quit tobacco and cannabis use simultaneously compared to mere psychoeducation. Because MI has shown promising effects as a motivational enhancement strategy for cannabis users [33], we additionally hypothesised that this intervention would outperform the effectiveness of the normative feedback intervention (H3).
Furthermore, this study aimed to evaluate the three interventions as they pertained to secondary outcome variables, i.e. the frequencies of tobacco and cannabis use. We had the same hypotheses for these outcomes as those explained above."

3b) CONSORT: Important changes to methods after trial commencement (such as eligibility criteria), with reasons

No changes were made.

3b-i) Bug fixes, Downtimes, Content Changes

Not relevant. While several bug fixes occurred prior to participant recruitment, they did not occur after recruitment commenced.

4a) CONSORT: Eligibility criteria for participants

"The inclusion criteria for study participation included any tobacco use during the past four weeks and any cannabis use during the past six months. As implicit inclusion criteria, participants had to speak German and be computer literate. There were no age restrictions or other exclusion criteria. "

4a-i) Computer / Internet literacy

"The inclusion criteria for study participation included any tobacco use during the past four weeks and any cannabis use during the past six months. As implicit inclusion criteria, participants had to speak German and be computer literate. There were no age restrictions or other exclusion criteria. "

4a-ii) Open vs. closed, web-based vs. face-to-face assessments:

"Recruitment for the present study ran parallel to recruitment for a feasibility study of the above-mentioned smoking cessation course for co-smokers of tobacco and cannabis. This was conducted in Zurich and a neighbouring city. The recruitment strategy has been described in the publication on the course development [21]. Briefly, recruitment was carried out online and offline. First, a press release about the course was issued, which resulted in several reports in local newspapers and on radio and TV stations. Furthermore, brochures and leaflets were sent to counselling centres for addiction prevention and treatment, psychiatrists, and health (care) centres in the canton of Zurich and in the bordering cantons. Additionally, two social media platforms and a teaser in the online edition of a popular free newspaper were used for recruitment. All of these referred to the start page of the website for more information."

"Participants who provided informed consent for the follow-up assessment could indicate whether they wanted to answer the follow-up questionnaire online or over the phone."

For the follow-up assessment, participants were contacted after eight weeks via their chosen mode (i.e., via email including a link to the online questionnaire or via telephone). Those who preferred to answer the questionnaire online received an email reminder after about two weeks if they had not yet completed the online questionnaire. Those who chose the telephone questionnaire were contacted up to ten times"

4a-iii) Information giving during recruitment

"If the users met these criteria, they were informed about the opportunity to participate in a study aiming to improve the website's information offerings."

No English version of the informed consent available.

4b) CONSORT: Settings and locations where the data were collected

The interventions were fully web-based and the pre- and post-assessments were conducted online, too.

"For the follow-up assessment, participants were contacted after eight weeks via their chosen mode (i.e., via email including a link to the online questionnaire or via telephone)."

4b-i) Report if outcomes were (self-)assessed through online questionnaires

The baseline and post-intervention assessments were conducted online.

The 8-week follow-up assessment was conducted online or over the phone: "Participants who provided informed consent for the follow-up assessment could indicate whether they wanted to answer the follow-up questionnaire online or over the phone."

"For the follow-up assessment, participants were contacted after eight weeks via their chosen mode (i.e., via email including a link to the online questionnaire or via telephone). Those who preferred to answer the questionnaire online received an email reminder after about two weeks if they had not yet completed the online questionnaire. "

4b-ii) Report how institutional affiliations are displayed

The logos of the research institute and of two addiction treatment centers involved in the project were displayed at the homepage.

5) CONSORT: Describe the interventions for each group with sufficient details to allow replication, including how and when they were actually administered

5-i) Mention names, credential, affiliations of the developers, sponsors, and owners

"Due to this finding, the authors developed three brief online interventions to enhance co-smokers' awareness of the relationship between the substances as well as their readiness to simultaneously quit each substance."

5-ii) Describe the history/development process

In-house testing was conducted for approximately two weeks following the program's development

5-iii) Revisions and updating

This is the first version of the intervention. No major changes to the program were made over the course of the study, and currently there are no plans to
modify the intervention.

5-iv) Quality assurance methods

To increase accuracy of information, the study employed validated measures that were suitable for online implemenation. We also assured participants that all information obtained is confidential and used ananymous codes to match the data that participants provided during the intervention session and the follow-up assessment.

5-v) Ensure replicability by publishing the source code, and/or providing screenshots/screen-capture video, and/or providing flowcharts of the algorithms used

The website is noted in the paper. Some screenshots of the interventions are included in the manuscript. Detailed flow-charts of the interventions (in German) are also available from the first author on request

5-vi) Digital preservation

The website can be viewed at http://i-cut.ch/

5-vii) Access

"The interventions were integrated within a German-language website (www.i-cut.ch), which, besides the interventions, contained information about an integrative group cessation course for co-smokers of tobacco and cannabis. This cessation course is evaluated in a separate study (ISRCTN15248397).
Participants could enter the current study in one of two ways. First, they could enter it directly from the start page of the website, where participants could choose between "getting more information about the course" and "learning more about my use of tobacco and cannabis". They were then directed to the course information pages or to the intervention session, respectively. We chose the cover term "learning more about my use of tobacco and cannabis" for the intervention session to attract co-smokers who were not seeking treatment. The second way to enter the study was to switch there from the course information pages by clicking a teaser that was displayed on the right side of each information page. It was also labelled "learning more about my use of tobacco and cannabis" (Figure 1). Conversely, participants could switch from the intervention session to the course information pages by clicking a hyperlink ("register now for the tobacco and cannabis cessation course"). This hyperlink was present on every page of the intervention, and the participants who clicked on it were directed to the course information pages and dropped out of the present study. Figure 2 shows a sample page of the intervention and the hyperlink.
"Recruitment for the present study ran parallel to recruitment for a feasibility study of the above-mentioned smoking cessation course for co-smokers of tobacco and cannabis. This was conducted in Zurich and a neighbouring city. The recruitment strategy has been described in the publication on the course development [21]. Briefly, recruitment was carried out online and offline. First, a press release about the course was issued, which resulted in several reports in local newspapers and on radio and TV stations. Furthermore, brochures and leaflets were sent to counselling centres for addiction prevention and treatment, psychiatrists, and health (care) centres in the canton of Zurich and in the bordering cantons. Additionally, two social media platforms and a teaser in the online edition of a popular free newspaper were used for recruitment. All of these referred to the start page of the website for more information.
To maximise the response rates, study participants were also offered the opportunity to participate in a lottery for three vouchers valued at 300, 200, or 100 Swiss Francs after they completed the first session, including the second measurement. Additionally, a second lottery for the same values served as an incentive to participate in the follow-up measurement."

5-viii) Mode of delivery, features/functionalities/components of the intervention and comparator, and the theoretical framework

"Personalised, normative feedback is one motivational technique that can be applied to web-based interventions for substance use. Based on the social norms approach [25], such interventions typically include self-assessment sections and feedback sections in which the participants' behaviour is compared to a reference sample. The overestimation of substance use by others is common and is positively associated with one's own use [26]. Web-based social norm interventions use this association and aim to correct the participants' erroneous perceptions. Mostly studied among college students and targeting alcohol use, web-based norms approaches for interventions have yielded promising results [27].
A further established technique for building motivation is motivational interviewing (MI), which uses a client-centred, directive counselling style to explore and reduce ambivalence and increase the intrinsic motivation for change [28]. Brief face-to-face interventions based on MI have been found to be effective in reducing cannabis use [29] and may assist in smoking cessation [30]. MI in web-based interventions is usually applied as a chat-intervention but is not fully automated. However, the first promising results of fully automated MI have recently been revealed by a computer-based intervention targeting perinatal drug use [31]."
"General Information and Technological Background
Participation in the interventions was free, and access was open for every eligible participant. The delivery of interventions was fully automated. The open source software LimeSurvey (Version 1.91) was used to program the survey and the interventions.
As described below, the interventions varied in the extent to which they were interactive with the participants. Additionally, the interventions differed in the way in which each was tailored to the responses that the participants had given during the baseline assessment and during the interventions themselves.
Intervention 1: Normative Feedback (NF)
The first intervention contained a combination of self-assessment and personalised normative feedback (NF). It consisted of three sections, including one each for tobacco use, cannabis use, and co-smoking. In the first and second sections, participants began by completing a questionnaire (the Fagerstrom Test of Nicotine Dependence, FTND [34,35], and the Cannabis Use Disorder Identification Test, CUDIT [36], respectively). Participants received feedback following each questionnaire. Feedback was individually tailored to participants using an algorithm based on the results from the FTND, the CUDIT, and the baseline data. Based on the social norms approach, each participant's reported frequency of smoking was presented in relation to the normative data from Swiss community samples. Afterwards, participants received feedback about their questionnaire scores and whether their responses met criteria for dependency (FTND) and/or problematic use (CUDIT), respectively. Explanations of "cigarette dependence" and "problematic cannabis use" were also given. Each substance-specific section concluded with brief recommendations for cessation or moderation of use. In addition, at the end of the intervention, information was provided that simultaneously accounted for the participant's use patterns of tobacco and cannabis. Participants who regularly smoked both tobacco and cannabis were informed about the group cessation course and referred to the end of the post-intervention assessment for further information. Participants who used either just one of the substances or both less regularly received contact details for the appropriate consulting services. Table 1 presents examples of translated feedback.
Intervention 2: Motivational Interviewing (MI)
The second intervention was based on the principles of motivational interviewing (MI). It was highly interactive and tailored to the participant, and it used a selection of MI techniques that could be adapted to a web-based intervention, such as open-ended questions, affirmative feedback, and periodic summaries. The aim of this intervention was to promote participants' self-reflective thinking about their own smoking behaviour and intentions to change it and to enhance their self-confidence in the ability to change. This was done in different tasks, such as decisional balance tasks, in which participants wrote down personal pros and cons of stopping tobacco use, cannabis use, or both simultaneously (Figure 2). Participants were also asked to write down what advice they would give to a co-smoking friend and to indicate their confidence in successfully stopping tobacco, cannabis, or both simultaneously on a confidence ruler. Participants received feedback, including a brief summary of their indicated change in self-confidence and a brief informational text about the simultaneous cessation of tobacco and cannabis use. To further enhance their self-confidence, participants were asked to list any behaviour that they had successfully changed in the past and to write down the names of persons in their network who could provide some level of social support during an attempt to quit smoking. Participants who, at baseline, had low levels of motivation to quit smoking and cannabis simultaneously received a further task.
Intervention 3: Psychoeducation (PE)
The third intervention was the active control group and provided psycho-educational information (PE) about tobacco and cannabis use. The information was thematically subdivided into smaller subsections. Participants had to read the sections in sequential order. Several terms and concepts that some readers may not know (e.g., "carbon monoxide") were explained in a small text box that appeared when mousing over the word of interest (Figure 4). The PE intervention started with an explanation of the association between the two substances with regard to the initiation and cessation of their use, their linking mechanisms, and the potential health consequences of their co-use. The next chapter contained information about the short- and long-term consequences of tobacco use, tobacco dependence, and the cessation of tobacco use and was followed by an analogous chapter on cannabis. The final chapter provided information about changing smoking behaviour and addressed smoking reduction versus abstinence, the simultaneous cessation of tobacco and cannabis use, and support during the cessation process. At this point, the group cessation program was mentioned and participants were referred to the end of the post-intervention assessment to receive further information. "

5-ix) Describe use parameters

"Overall, participants remained in the intervention sessions for an average of 25.5 minutes (SD = 33.0), including the baseline and post-intervention assessments. While the participants in the NF condition finished the session after M = 17.0 minutes (SD = 9.1) on average, participants in the PE (M = 28.4, SD = 38.4) and the MI (M = 28.9, SD = 41.6) interventions stayed significantly longer ($F_{2,322}$ = 4.7, P = .01)."

5-x) Clarify the level of human involvement
"The delivery of interventions was fully automated."
For the trial: "For the follow-up assessment, participants were contacted after eight weeks via their chosen mode (i.e., via email including a link to the online questionnaire or via telephone). "
5-xi) Report any prompts/reminders used
No prompts/reminders to use the application, only for the follow-up assessment: "Those who preferred to answer the questionnaire online received an email reminder after about two weeks if they had not yet completed the online questionnaire. Those who chose the telephone questionnaire were contacted up to ten times. "
5-xii) Describe any co-interventions (incl. training/support)
"The interventions were integrated within a German-language website (www.i-cut.ch), which, besides the interventions, contained information about an integrative group cessation course for co-smokers of tobacco and cannabis. This cessation course is evaluated in a separate study (ISRCTN15248397). "
6a) CONSORT: Completely defined pre-specified primary and secondary outcome measures, including how and when they were assessed
"The primary outcome measure was participants' readiness to quit the use of tobacco and cannabis simultaneously. Readiness was measured at all three time points by the question "To what extent are you ready to quit tobacco and cannabis simultaneously?" Participants indicated their readiness on a ruler ranging from 1 ("not at all") to 10 ("very much"). The item was designed based on the contemplation ladder [22], which is especially suited to measure early stages of readiness. In addition, a comparison of the readiness ruler to other measures of motivation to change revealed its good concurrent and predictive validity and its superior clinical utility when its brevity and ease of administration are considered [37].
Secondary outcomes included the self-reported frequency of tobacco and cannabis use at baseline (t0) and at the 8-week follow-up (t2). The frequency of tobacco use was defined as the daily amount of cigarettes smoked during a typical smoking day, corrected for the number of smoking days during the past month. The frequency of cannabis use in the past week was assessed using 7-day timeline follow-back question [38]. "
6a-i) Online questionnaires: describe if they were validated for online use and apply CHERRIES items to describe how the questionnaires were designed/deployed
The measure of the primary outcome was a single item (a readiness ruler) rather than a questionnaire. Tthe best of our knowledge, the readiness ruler has not yet been validated for online use.
6a-ii) Describe whether and how "use" (including intensity of use/dosage) was defined/measured/monitored
Use was defined as the duration of the intervention session (including the baseline and post-intervention assessment). This was recorded by the software limesurvey.
6a-iii) Describe whether, how, and when qualitative feedback from participants was obtained
Not applicable. No qualitative feedback was obtained.
6b) CONSORT: Any changes to trial outcomes after the trial commenced, with reasons
No changes.
7a) CONSORT: How sample size was determined
7a-i) Describe whether and how expected attrition was taken into account when calculating the sample size
The required sample size was 246. We recruited 326 participants in order to account for attrition.
7b) CONSORT: When applicable, explanation of any interim analyses and stopping guidelines
Not applicable.
8a) CONSORT: Method used to generate the random allocation sequence
"Once the baseline measurement (t0) was completed, participants were randomly assigned to one of three possible interventions. "
This was done in a fully atuomated manner by the software used for the assessments and the interventions (limesurvey).
8b) CONSORT: Type of randomisation; details of any restriction (such as blocking and block size)
No restriction of randomisation. The probability of allocation was 33.3% for each condition.
9) CONSORT: Mechanism used to implement the random allocation sequence (such as sequentially numbered containers), describing any steps taken to conceal the sequence until interventions were assigned
"Once the baseline measurement (t0) was completed, participants were randomly assigned to one of three possible interventions. "
This was done in a fully atuomated manner by the software used for the assessments and the interventions (limesurvey).
10) CONSORT: Who generated the random allocation sequence, who enrolled participants, and who assigned participants to interventions
The random allocation sequence was computer generated (by the software limesurvey).
11a) CONSORT: Blinding - If done, who was blinded after assignment to interventions (for example, participants, care providers, those assessing outcomes) and how
11a-i) Specify who was blinded, and who wasn't
The participants did not know that different interventions were tested and were therefore "blinded".
The researachers who conducted the follow-up-assessment were blinded.
11a-ii) Discuss e.g., whether participants knew which intervention was the "intervention of interest" and which one was the "comparator"
No, participants did not knwo which was the intervention of interest becasue they were not informed about the existence of different interventions during their participation.
11b) CONSORT: If relevant, description of the similarity of interventions
see section "Interventions"
12a) CONSORT: Statistical methods used to compare groups for primary and secondary outcomes
"To analyse the primary and secondary outcome variables, we used generalised estimating equations (GEE) that consider the correlated nature of repeated measures. "
12a-i) Imputation techniques to deal with attrition / missing values
"Using the Amelia II multiple imputation package of the R software environment for statistical computing, Version 2.15.3 [39], we imputed 20 datasets. In a simulation study using data from an online self-help program for problem drinkers, Amelia II outperformed other methods of multiple imputation [40]. Hypotheses tests were performed using each data set separately and were pooled afterwards (intention-to-treat analysis)."
12b) CONSORT: Methods for additional analyses, such as subgroup analyses and adjusted analyses
"we also performed complete case analyses considering only participants who provided data at all three assessments.""

RESULTS

13a) CONSORT: For each group, the numbers of participants who were randomly assigned, received intended treatment, and were analysed for the primary outcome
"325 (22.1%) provided informed consent, completed the baseline assessment, and could therefore be randomised into one of the intervention groups."
In the ITT analyses, all 325 participants were analysed.
13b) CONSORT: For each group, losses and exclusions after randomisation, together with reasons
See stuy flow chart (Figure 5).

13b-i) Attrition diagram
See stuy flow chart (Figure 5).
14a) CONSORT: Dates defining the periods of recruitment and follow-up
"The data was collected between January and November 2012."
14a-i) Indicate if critical “secular events” fell into the study period
Not applicable.
14b) CONSORT: Why the trial ended or was stopped (early)
The trial was not stopped early.It was stopped because the sufficient sample size was reached.
15) CONSORT: A table showing baseline demographic and clinical characteristics for each group
See Table 2.
15-i) Report demographics associated with digital divide issues
Age and gender are reported. Computer literacy was an implicit inclusion criterion.
16a) CONSORT: For each group, number of participants (denominator) included in each analysis and whether the analysis was by original assigned groups
16-i) Report multiple “denominators” and provide definitions
Both ITT and complete case analyses were employed.
The paper reports on number of compliers, dropout rate, and duration of the intervention session (including t0- and t1-assessments).
16-ii) Primary analysis should be intent-to-treat
Both ITT and CCA were performed.
17a) CONSORT: For each primary and secondary outcome, results for each group, and the estimated effect size and its precision (such as 95% confidence interval)
see Results sections "Effects of the Intervention on Readiness to Simultaneously Quit Tobacco and Cannabis Use" and "Effects of the Intervention on Secondary Outcomes "
17a-i) Presentation of process outcomes such as metrics of use and intensity of use
We collected information on information session length.
17b) CONSORT: For binary outcomes, presentation of both absolute and relative effect sizes is recommended
No binary outcomes were analysed.
18) CONSORT: Results of any other analyses performed, including subgroup analyses and adjusted analyses, distinguishing pre-specified from exploratory
"To determine the robustness of our results to the analytic strategy, we also performed complete case analyses considering only participants who provided data at all three assessments."
Other subgroup-analyses were not performed.
18-i) Subgroup analysis of comparing only users
"To determine the robustness of our results to the analytic strategy, we also performed complete case analyses considering only participants who provided data at all three assessments."
Other subgroup-analyses were not performed.
19) CONSORT: All important harms or unintended effects in each group
No harms or unintended effects were observed within the study.
19-i) Include privacy breaches, technical problems
Privacy breaches and significant techinical problems did not occur during the course of the trial.
19-ii) Include qualitative feedback from participants or observations from staff/researchers
No qualitative feedback was collected.

DISCUSSION

20) CONSORT: Trial limitations, addressing sources of potential bias, imprecision, multiplicity of analyses
20-i) Typical limitations in ehealth trials
The limitations section addresses potential biases due to attrition, study design, and differences between the interventions regarding their length.
21) CONSORT: Generalisability (external validity, applicability) of the trial findings
21-i) Generalizability to other populations
Generalizability to a general Internet population is not possible because the interventions are tailored for co-smokers of tobacco and cannabis.
21-ii) Discuss if there were elements in the RCT that would be different in a routine application setting
The differences from real-world use involve completion of assessments.
22) CONSORT: Interpretation consistent with results, balancing benefits and harms, and considering other relevant evidence
22-i) Restate study questions and summarize the answers suggested by the data, starting with primary outcomes and process outcomes (use)
The Discussion opens by discussing the research hypotheses. A brief discassion of the use is also included in this section.
22-ii) Highlight unanswered new questions, suggest future research
This is addressed in various paragraphs of the paper.

Other information

23) CONSORT: Registration number and name of trial registry
Current Controlled Trials, ISRCTN56326375
24) CONSORT: Where the full trial protocol can be accessed, if available
http://www.controlled-trials.com/ISRCTN56326375
25) CONSORT: Sources of funding and other support (such as supply of drugs), role of funders
“The Swiss Tobacco Control Fund provided financial support for this study (grant number 11.002932).”
X26-i) Comment on ethics committee approval
"The study was designed in accordance with the Helsinki declaration and was approved by the ethics committee of the Canton of Zurich, Switzerland (approval number: KEK-StV-Nr. 23/11, June 27, 2011, and amendment for the internet-based intervention, November 11, 2011). The study is registered at current controlled trials (http://www.controlled-trials.com, ISRCTN56326375)."
x26-ii) Outline informed consent procedures

X26-iii) Safety and security procedures

Anonymous data collection: "Participants of the study were then instructed to create an anonymous but personal identification code according to a rule combining certain letters of the parents’ names and their own date of birth. The same procedure was applied at the follow-up assessment in order to allow for linking of the data of the different assessments."

X27-i) State the relation of the study team towards the system being evaluated

“The authors were involved in the development of the interventions.”
